# Academic education of midwives in Germany (part 2): Opportunities and challenges for the further development of the profession of midwifery. Position paper of the Midwifery Science Committee (AHW) in the DACH Association for Medical Education (GMA)

**DOI:** 10.3205/zma001687

**Published:** 2024-06-17

**Authors:** Sabine Striebich, Nicola H. Bauer, Kirsten Dietze-Schwonberg, Melita Grieshop, Annette Kluge-Bischoff, Birgit-Christiane Zyriax, Claudia F. Plappert

**Affiliations:** 1 Martin Luther University Halle-Wittenberg, University Medicine Halle, Medical Faculty, Institute of Health and Nursing Science, Halle (Saale), Germany; 2 University of Cologne and University Hospital Cologne, Medical Faculty, Institute for Midwifery Science, Cologne, Germany; 3 University Hospital of Johannes Gutenberg University Mainz, Mainz, Germany; 4 Protestant University of Applied Sciences Berlin, Berlin, Germany; 5 University of Augsburg, Medical Faculty, Augsburg, Germany; 6 University Medical Center Hamburg-Eppendorf, Midwifery Science – Health Services Research and Prevention,Institute for Health Services Research in Dermatology and the Nursing Professions (IVDP), Hamburg, Germany; 7 University of Tübingen, Institute of Health Science, Tübingen, Germany

**Keywords:** academisation, health professionals, midwives, discipline development

## Abstract

The objective of academic training is to prepare midwives as independent healthcare professionals to make a substantial contribution to the healthcare of women in their reproductive years as well as to the health of their children and families. This article therefore describes the professional and educational requirements derived from the legal midwifery competencies within the new midwifery act. Furthermore, it identifies the conditions that need to be established to enable midwives in Germany to practise to their full scope in compliance with statutory responsibilities.

Educational science, academic efforts, policymaking and accompanying research should work in synergy. This in turn enables midwives to achieve the maximum scope of their skills, with the objective of promoting physiological pregnancies and births. Consequently, it can strengthen early parenthood in alignment with the national health objectives of “health around childbirth”.

The academisation of the midwifery profession presents a profound opportunity for professional development in Germany. It is essential that midwives receive training based on the principles of educational science and care structures that are yet to be developed. This can enable them to perform within the wide range of their professional tasks to the highest standards, thereby ensuring the optimal care of their clients. Moreover, there is a chance to implement sustainable improvements in healthcare provision for women and their families during the reproductive phase and the period of parenthood in Germany.

## Abstract (de)

Die akademische Bildung soll Hebammen in die Lage versetzen, als selbstständige Gesundheitsfachpersonen einen umfassenden Beitrag in der Gesundheitsversorgung von Frauen in der reproduktiven Lebensphase, ihrer Kinder und ihrer Familien zu leisten. Im vorliegenden Artikel wird dargelegt, welche fach- und bildungswissenschaftlichen Anforderungen sich aus den Kompetenzzielen des neuen Berufsgesetzes ergeben, und aufgezeigt, welche Voraussetzungen geschaffen werden müssen, damit Hebammen in Deutschland gemäß dem gesetzlich definierten Aufgabenspektrum tätig sein können.

Bildungswissenschaftliche Anstrengungen und versorgungspolitische Maßnahmen einschließlich Begleitforschung sollten Hand in Hand gehen, um Hebammen in die Lage zu versetzen, ihre Kompetenzen zur Förderung physiologischer Schwangerschaften und Geburten und zur Stärkung der frühen Elternschaft gemäß dem Nationalen Gesundheitsziel „Gesundheit rund um die Geburt“ bestmöglich einzubringen. Dabei stellen Maßnahmen zur Gestaltung effektiver interprofessioneller Zusammenarbeit mit anderen Gesundheitsberufen gemäß den jeweiligen Berufsaufgaben und -rollen im ambulanten und im stationären Sektor und an den Schnittstellen einen Kernaspekt dar. Die wissenschaftliche Nachwuchsförderung von Master, über Promotion bis Habilitation muss ausgebaut werden.

Die Akademisierung des Hebammenberufs eröffnet große Chancen der Professionsentwicklung. Damit Hebammen gemäß ihres breiten beruflichen Aufgabenspektrums tätig sein können, werden bildungswissenschaftlich fundierte Lehrkonzepte sowie geeignete, neu zu entwickelnde Versorgungsstrukturen benötigt. Das birgt das Potenzial, die gesundheitliche Versorgung von Frauen und Familien in der reproduktiven Lebensphase des Elternwerdens in Deutschland nachhaltig zu verbessern.

## 1. Background

With the adoption of the new professional laws in 2019 and 2020 (Midwifery Act [https://www.gesetze-im-internet.de/hebg_2020/BJNR175910019.html] and Midwifery Study and Examination Ordinance [https://www.gesetze-im-internet.de/hebstprv/BJNR003900020.html]), the transfer of the midwifery profession to the academic level in Germany has gained momentum. The neighbouring countries of Austria and Switzerland had already undergone the same process 15 years earlier. However, the development of the profession and the existing regulations governing midwifery practice in this country differ significantly from those in Austria and Switzerland.

1. **Developments in the midwifery profession in Austria – a brief overview**
  In Austria, the invoicing of freelance, independent services with health insurance providers is contingent upon the individual registration with their health insurance fund. Nevertheless, in recent years, the recruitment and re-recruitment of midwives to these roles has often been challenging due to the lack adequate financial funding and the inflexibility of working schedules. In many regions, this has resulted in a significant number of independent midwives whose services are available and largely financed privately by women and families. This development has led to the emergence of a two-tier midwifery care system, which politicians have attempted to address through the introduction of a new comprehensive contract. This contract ensures the provision of enhanced financial funding, greater operational flexibility for the interaction of midwives with health insurance funds, as well as a second appointment with a midwife during the pregnancy period. Nevertheless, with the exception of two consultations with midwives, antenatal care in Austria is the exclusive domain of obstetricians. Consequently, the core competencies of freelance midwives remain significantly constrained. The establishment of so-called primary care centres, as currently being implemented, has the potential to strengthen the role of midwives in primary healthcare. The revised Primary Care Act explicitly identifies midwives as an essential component of care in the 150 primary care centres planned for establishment by 2025.
2. **Developments in the midwifery profession in Switzerland – a brief overview**
  In Switzerland, as in Germany and Austria, midwives may work in an employment, as affiliated freelance midwives in hospitals, or they are completely self-employed. In addition to the existing competencies and qualifications, universities also offer a range of further education qualifications. These include the Certificate of Advanced Studies (CAS, 10-15 ECTS), the Diploma of Advanced Studies (DAS, 30 ECTS) and the Master of Advanced Studies (MAS, 60 ECTS). A CAS degree is the academic prerequisite for the role of expert within university hospitals. Alternatively, an MAS or MSc is necessary for the role of midwifery expert. Specialist midwifery experts are required to hold at least an MSc degree. Therefore, MSc programmes designed for students wishing to become Advanced Practice Midwives provide the necessary training. This profile is currently in development by universities in collaboration with practitioners (Conference for Midwifery Profession within Health Conference and Swiss Association of Midwives, 2021).

The aim of academic education is to enable midwives to think critically about their actions as “reflective practitioners”. This should facilitate the development of their own research activities and academic career paths, as well as the shaping of responsible interprofessional collaboration [https://www.gesetze-im-internet.de/hebg_2020/BJNR175910019.html]. Midwives are qualified to practise “independent and comprehensive midwifery work in the inpatient and outpatient sector” and to do so in accordance with the “generally recognised standard of midwifery, medical and other scientific knowledge on the basis of professional ethics” (Midwifery Act (HebG) 2019, study objective §9) [https://www.gesetze-im-internet.de/hebstprv/BJNR003900020.html], with further details on §9 available in attachment 1 , table S1. 

The legally defined competencies for graduates of a Bachelor's degree programme [1] (see attachment 1 , table S2) in the Midwifery Study and Examination Ordinance (HebStPrV 2020), Appendix 1, establish the responsibility of midwives for planning the healthcare of women and trans*, inter and non-binary people who are pregnant and give birth to a child in central Europe. The aforementioned competencies extend to encompass the reproductive phase of life, encompassing family planning, pregnancy, birth, the postpartum period, and the end of the child's first year of life or the end of breastfeeding (“Midwifery care continuum” [in Germany called “Betreuungsbogen”] [2]). In the following text, the gender identity of individuals who use midwifery services is not explicitly labelled [3]; women is used as a general term to refer to all individuals in this context.

The ninth National Health Goal (NHG) “Health around childbirth” (2017) [4] stipulates that obstetric care in Germany should be more woman- and family-centred and health-oriented in future. The recognition of risks and burdens for pregnant women and parents-to-be at an early stage is of paramount importance, with resources being promoted and tailored assistance offered. Moreover, the objective should be to facilitate the attainment of the highest possible standard of health for pregnant women and their infants, with a minimum number of well-founded interventions. Furthermore, it is essential to strengthen parents in the early phase of parenthood and to promote the healthy development of families [4]. The regular and continuous involvement of midwives throughout the care pathway (referred to as “midwifery-led care”) is an evidence-based global strategy to improve women’s health [5]. It can improve numerous key health outcomes for mother and child [6] and reduces the number of interventions [7]. The implementation of preventative measures for pregnant women with regard to diet and exercise is of essential significance in reducing long-term health risks [8]. Yet, pregnancy is regarded as for adopting a healthy lifestyle throughout life [9], as it shapes the health of the foetus for life, a process referred to as “foetal programming” [8]. 

## 2. Midwife care to promote physiological pregnancies

Pregnancy and birth have a rectifying impact on the health behaviours of families, with implications for nutrition, physical activity, dental care and vaccination. These experiences serve as a foundation for long-term physical and mental health outcomes [10].

### 2.1. Antenatal care to improve the health literacy of women and families

Antenatal care, as recommended by the NHG, is designed to enhance the health competencies of women and expectant parents, with due consideration of their individual requirements, constraints and potential risk factors. Midwives provide evidence-based information and contribute to a healthy lifestyle through personal counselling. Despite the potential advantages, the representation of midwives in antenatal care in Germany is currently marginally low [11]. A particularly notable deficiency in midwife support is observed in areas with high birth rates and poor social circumstances as evidenced by data from Hamburg [12]. Midwives are expected to positively influence the health behaviour of pregnant women and parents-to-be [13], such as through advice on nutrition and exercise to prevent gestational diabetes [14]. Therefore, some federal states have incorporated counselling on contraception, family planning and sexual health into their midwifery professional code of conduct regulations. The incorporation of this content into the curricula of degree programmes and the structural integration of this activity within the healthcare system could have a beneficial impact on health literacy and the sexual and reproductive health of the population [15]. Midwives can obtain additional qualifications to enable them to provide support to pregnant women with specific needs through further training. In England, for instance, the role of the specialist mental health midwife [16] has been established, with the objective of contributing to effective perinatal healthcare for women with mental health problems. 

### 2.2. Midwife care for support in decision-making situations

Midwives provide information and advice on decision-making situations where there are multiple equally valid options, such as the choice of place of birth. The concept of shared decision-making (SDM) [17] is particularly relevant in this context and is also referenced in the midwifery contract [13]. In order to present all options with their respective advantages and disadvantages in an intelligible manner, midwives must possess the requisite competencies. These include the ability to ascertain the values and aspirations of the other individual, to collaborate in the joint determination of a course of action, and to review this decision. Furthermore, they must possess competencies in risk communication, as well as an understanding of the roles and responsibilities within an interprofessional team. These professional skills are essential for the effective support of decision-making processes [18]. Accordingly, the acquisition of counselling skills in SDM should be an integral aspect of the degree course.

### 2.3. Midwifery care in midwifery centres

Midwives provide healthcare services in a variety of settings, including as lone workers and within midwifery centres or departments (also known as “midwife-led units”, MLU [so called “hebammengeleitete Einrichtung, hgE”]). This setting allows for the provision of continuous midwifery-led care [5] by a dedicated team of midwives for a greater number of women than was the case previously. Furthermore, group programmes could be implemented that have been demonstrated to be beneficial and resource saving, such as those employed in antenatal care [19]. These MLUs could be sited either externally or on the grounds of maternity hospitals. Additionally, they may encompass out-of-hospital-births, and a postnatal outpatient clinic. This concept could represent a safe and expandable approach to primary care in Germany, as evidenced by an analysis of data from North Rhine-Westphalia [20]. Furthermore, these centres could be of significant benefit as training sites for students.

## 3. Midwifery care to promote physiological births

### 3.1. Midwife-led births in hospitals

In Germany, obstetrics is a domain reserved for midwives and doctors (HebG § 4). While midwives can attend physiological births independently and autonomously, doctors are obliged to consult midwives for every birth. However, in the event of complications, midwives are obliged to consult a doctor. The GKV-Spitzenverband’s (National Association of Statutory Health Insurance Funds) catalogue of criteria for midwife-led births outside of hospitals [21] outlines the findings and risks that rules out the option of out-of-hospital birth. However, in hospitals, births are typically attended by a team of both midwives and doctors. In the absence of risk factors, a midwife-led birth in a “midwife-led delivery suite” (MLDS) [22] is currently possible in approximately 36 of the approximately 621 existing maternity units (as of 2024) [23], although the exact number of MLDSs is unknown due to the lack of protection afforded to the term. Analyses have demonstrated that births in MLDSs are associated with a reduced number of interventions, as well as high levels of satisfaction among parents [20]. It is to be expected that in future, maternity care in hospitals will be further concentrated in large units. Moreover, small, local maternity units close to parents homes, previously offering maternity and birth services to pregnant women with low-risks pregnancies, will be closed [24], [25]. This again will result in a capacity bottleneck in large hospitals, which will then be required to care for women with different risk profiles to a greater extent than previously. Consequently, risk-adapted care concepts for hospital births will be required in the future. The reform of hospital care proposes the categorisation of hospitals into three distinct care levels, designated as leveI I, II, and III [26]. The German Society for Midwifery Science (Deutsche Gesellschaft für Hebammenwissenschaft – DGHWi) [27] and the German Midwifery Association (Deutscher Hebammenverband – DHV) [28] consider the routine anchoring of midwife-led birth at all levels of care to be important to realise risk-adapted care.

### 3.2. Counselling services by midwives and interprofessional teams 

In accordance with the NHG, midwife-led consultations in hospitals should henceforth serve as a regular point of contact for pregnant women. This is not only to fulfil the legal obligation to provide information on medical interventions in accordance with BGB §630e (German Civil Code), but also to provide information on midwife assistance during birth, for example on non-pharmacological measures for pain relief. It is recommended that all pregnant women receive this type of counselling and information on a regular basis. Moreover, these services should be complemented by evidence-based interprofessional education and counselling for planned medical interventions, such as induction of labour, birth planning for multiple births, epidural anaesthesia, and planned caesarean section, as well as breech presentation births. Counselling for rare events, such as accompanying a trans man during his birth [29] or after the birth of an intersex child [30], should also be included. Finally, Early Assistance services, such as “Baby Guides” (in German “Babylotsen”), offered to vulnerable families up to three year after childbirth [31], should be implemented. 

## 4. Promotion of interprofessional collaboration

In collaboration with doctors, midwives shape the culture of the workplace within the hospital environment. A progressive working culture and a coherent attitude of hospital staff are associated with a lower rate of primary caesarean sections (planned caesareans sections before onset of labour) [32] and greater job satisfaction among hospital staff. This again can counteract the shortage of skilled workforce, as evidenced by experience from Sweden [33]. The realisation of the NHG is contingent upon the promotion of interprofessional collaboration. This entails the establishment of respectful communication and an adequate understanding of roles, shared values and good team cooperation [34]. This is because for women giving birth, a trusting relationship with obstetric staff and continuous support are particularly predictive of a high level of satisfaction with the birth experience [35].

The establishment of rules on the shared responsibilities of the various professions should be described in internal standard operating procedures (SOPs). These SOPs for instance, could describe how a woman-centred birth environment can be ensured in the hospital, including for women with risk factors [36]. Furthermore, they could describe how guidelines with novel and unfamiliar recommendations can be implemented in practice, such as intermittent fetal heart rate auscultation during birth [37]. It is well established that respect and acknowledgement from colleagues, as well as opportunities to shape the workplace and organisational and practical facilitations, can influence job satisfaction and promote job retention [38]. Therefore, interprofessional collaboration can be a significant factor in fostering greater respect and satisfaction.

## 5. Further development of scientific career pathways

The proportion of midwives within the healthcare professions with an academic degree is relatively high. However, there is a need for further development at the master’s level [39]. Currently, twelve three- to five-semester consecutive Master’s degree programmes in Germany offer a specialisation in midwifery and healthcare/nursing science, as well as health services research. Such programmes are typically accredited with 90 or 120 ECTS credits. The competence objectives are advanced midwifery practice (AMP) or research activities. Internationally, the concept of AMP is understood to encompass a range of extended fields of activity and roles for midwives [40]. In Germany, however, a comprehensive debate and consensus is required within the discipline regarding activities that midwives with an AMP qualification could undertake in the domains of care, education, and research. This again has the potential to ensure that they contribute to the optimisation of care, the improvement of learning in practice, and the management of clinical trials [41], [42]. Moreover, this discourse should be accompanied by a corresponding implementation strategy. There is evidence suggesting that a lack of scientific knowledge in practice is an important factor that hinders evidence-based care in Germany [43]. Those with a Master's degree in Midwifery in Germany could contribute to the development of clinical guidelines, lead interprofessional evidence panels or journal clubs [44][. Furthermore, they could generate standards for effective teamwork in emergencies and for inter-sectoral interface management. Finally, they could plan clinical care concepts for women with specific needs or risk factors, including pregnant women with fear of childbirth [45], [46], women with disabilities [47] or women who have given birth to a premature child or a neonate that [48]. Moreover, the creation of recommendations regarding the management of specific professional, ethical, or legal tasks undertaken by interprofessional teams is of great importance. Such tasks include providing information on episiotomy [49], [50] and offering expert counselling to pregnant women who are planning a vaginal breech birth [51]. Additionally, the implementation of peer reviews [52] and, finally, the introduction of basic ultrasound diagnostics [53] represent potential avenues for expanded midwifery practice that could alleviate the burden on other healthcare professions and enhance the quality of care.

For future lecturers, it is currently possible to pursue a Master’s degree programme in vocational education with a focus on health, nursing, general vocational education or medical education at 14 universities. These programmes are offered over a period of four to eight semesters and lead to a master of health professions education qualification [54].

The systematic support of young scientists (doctorate, habilitation) is essential, and should include doctoral programmes, and mentoring or postgraduate colleges at universities. This is of particular importance in the context of universities of applied sciences (UASs). In accordance with the recommendations of the Health Research Council [55], such support should be provided through cooperative doctorates or in the form of nation-wide, university-independent UAS doctoral centres or colleges.

The fact that approximately 50% of professorships in midwifery science could not be filled between 2017 and 2019 [40] illustrates the pressing need to encourage the advancement of young academics. The appointment of an individual lacking expertise in the relevant field of midwifery to a professorship carries the risk of losing sight of the specific subject reference in both teaching and research. Such decisions can again have a detrimental impact on the discipline’s long-term evolution and advancement [56].

It would be beneficial to develop professorships at universities further by giving the post holders a clinical area of responsibility in “client care” at the university hospital in future. This would be analogous to professorships in medicine, in order to anchor midwifery science as an independent clinical discipline, thus better interlink theory, and care practice [57]. The integration of universities into clinical care could facilitate the creation of new opportunities for practice guidance, supervision, research, and team development.

## 6. Midwifery science research

In compliance with the standards set forth in HebG §9 (3) [https://www.gesetze-im-internet.de/hebstprv/BJNR003900020.html], those who have obtained a Bachelor’s degree in midwifery are expected to demonstrate the ability to “explore areas of research [...] in line with the latest established knowledge”. Consequently, the programme encompasses the basics of scientific work, as well as both qualitative and quantitative research methods. 

The Scientific Commission of the German Science and Humanities Council (WR) [https://www.gesetze-im-internet.de/hebg_2020/BJNR175910019.html] identifies three principal areas of research in midwifery science: application-oriented research, translational research, and clinical research. Application-oriented research aims to generate evidence for interventions, while translational research systematically reviews the benefits of interventions and their transfer to routine care. Clinical research, in turn, encompasses the investigation of the efficacy and safety of interventions in clinical settings. In the Lancet series on the further development of midwifery research, projects to maintain and promote physiological processes, both in normal pregnancies and in cases of existing risks or complications, are considered a priority [58]. 

The development of a dedicated research profile in the health professions in Germany is an important task [55]. In order to facilitate the realisation of larger clinical studies, universities have established research infrastructures. To date, only a few public research tenders have been relevant for midwifery researchers. It is challenging for research proposals from midwifery science to compete with those from established research foci on cardiovascular diseases, cancer, or areas of basic research. This makes the development process more difficult. Consequently, the majority of midwifery research projects are currently smaller in scale, with some being funded by the universities' own resources.

The experience of Australia and New Zealand [59] indicates that university research networks that combine nursing and midwifery expertise can be beneficial in the preparation of research proposals [60]. This is particularly the case with regard to interpretative-hermeneutic studies that explore the views and experiences of participants or previously unexplored phenomena that are particularly relevant in the discipline [61].

## 7. Conclusion

As anticipated by experts in 2016 [62], the transition to academic training of the midwifery profession presents new opportunities for the professionalisation and further development of midwifery and midwifery science. In order to capitalise on these opportunities and prepare students for their professional roles in the most effective manner, it is essential to engage in continuous pedagogical and didactic work on teaching concepts and their evaluation, as well as curriculum development. 

Furthermore, it is recommended that further developments in practical midwifery be given curricular consideration in academic midwifery education. This should include the theory-practice transfer, the development of suitable examination formats such as case studies, OSCEs, reflection tasks or formats for Bachelor's theses. Additionally, interprofessional teaching programmes should be developed and used on a regular basis. In order to ensure comparability of Bachelor's degrees, it is necessary to agree upon educational objectives, define examination standards, operationalise the legally defined competence objectives and develop a core curriculum for midwives. The Board for Midwifery Science in the Association for Medical Education (GMA) provides the appropriate framework for this.

Concurrently, the discipline is obliged to assume an active role in the shaping of cultural change in the practice of maternity care in accordance with the NHG [4]. In the context of the forthcoming structural system change in hospital planning [26], there is an opportunity to define specific midwife job profiles in the outpatient and inpatient settings with the objective of promoting physiological pregnancy and birth, as well as bonding and breastfeeding – central goals of the NHG. This should include interprofessional care concepts in interface management for transitions between the outpatient and inpatient sectors [25]. It is independently necessary to consider the educational and research-related requirements in this context. The results of project evaluations should be checked for educational requirements to be addressed and incorporated into curriculum development.

It is recommended that the health competence of student midwives and their pedagogical and didactic knowledge be accorded a higher priority in order to enable midwives to provide health education and counselling. These professional activities are central according to the terms of the Midwifery Assistance Contract [13], to be carried out in a health-promoting and target group-oriented manner. It is necessary to develop educational and evaluation concepts for new complex interventions in midwifery, such as counselling on family planning, contraception or sexuality, or for basic ultrasound diagnostics. In order to achieve this, working groups could be established within the GMA for this purpose.

In order to enhance the transfer of theoretical knowledge to practical applications, universities should proactively engage in collaboration with practice supervisors. To assess the quality of the learning environment in practice placements, it is essential to develop multidimensional and valid instruments. Therefore, existing instruments, such as the Australian Midwifery Standards Assessment Tool (AMSAT) [63], should be translated and culturally adapted.

## Notes

### Authors’ ORCIDs

- Sabine Striebich: [0000-0002-0626-3552]
- Nicola H. Bauer: [0000-0002-7351-0832]
- Kirsten Dietze-Schwonberg: [0000-0001-7649-4754]
- Melita Grieshop: [0000-0002-3317-3501]
- Annette Kluge-Bischoff: [0000-0002-3317-3501
- Birgit-Christiane Zyriax: [0000-0002-5377-5956]
- Claudia F. Plappert: [0000-0003-1896-3959]

### Accepted

The position paper was adopted by the GMA executive board at May 72024.

## Acknowledgements

The authors of the position paper would like to thank the following experts for their advice during the completion of the manuscript:

- Prof. Anne Wiedermann, Chair of Midwifery Science, Faculty of Interdisciplinary Studies at Landshut University of Applied Sciences
- Prof. Dr. Susanne Grylka, Head of Research Institute for Midwifery Science and Reproductive Health at the Zurich University of Applied Sciences ZHAW
- Dr. Astrid Krahl, Head of MSc Midwifery at the Institute of Midwifery, Department of Health, ZHAW Zurich University of Applied Sciences

The authors thank Emine Babac for translation of the article.

## Competing interests

The authors declare that they have no competing interests. 

## Supplementary Material

Excerpts from the midwives act
