# Supplementary material for: Academic education of midwives in Germany (part 2): Opportunities and challenges for the further development of the profession of midwifery. Position paper of the Midwifery Science Committee (AHW) in the DACH Association for Medical Education (GMA)
Source: GMS J Med Educ. 2024 Jun 17;41(3):Doc32. doi: 10.3205/zma001687 (PMC11310788; doi:10.3205/zma001687)
Supplement: Excerpts from the midwives act [file JME-41-32-s-001.pdf]

Attachment 1: Excerpts from the midwives act

Table S1: *Aim of the midwifery degree programme according to §9 of the Midwifery Act [https://www.gesetze-im-internet.de/hebg_2020/__9.html]*

(1) The midwifery degree programme provides students with the professional and personal skills required for independent and comprehensive midwifery work in inpatient and outpatient settings. The programme is taught in accordance with scientific principles and methodology. Lifelong learning is viewed as an ongoing process of professional growth and development, with the recognition that continuous personal and professional improvement is essential.

(2) Midwifery work is carried out in accordance with the generally recognised standards of midwifery, medical and other scientific knowledge on the basis of professional ethics. Midwives take into account the special needs of people with disabilities and chronic disorders. They consider the specific life situation, the social, biographical, cultural and religious background, sexual preference and the phase of life of the women and families to be cared for. They support their independence and respect their right to autonomy.

(3) The midwifery degree programme should enable midwives to...

1. plan, manage and organise highly complex care processes including prevention and health promotion measures in the field of midwifery on the basis of science-based and science-oriented decisions,
2. to be able to access research areas of midwifery science at the latest state of the art and to transfer research-based problem solutions as well as new technologies into professional action and to recognise professional training and further education needs,
3. critically and analytically analyse both theoretical and practical knowledge, and develop and implement innovative, science-based solutions to improve their own professional field of activity and
4. participate in the development of quality management concepts, risk management concepts, guidelines and expert standards.

(4) The midwifery degree programme should additionally enable student midwives

1. to carry out the following tasks independently:

a) to determine a pregnancy,

b) monitor the physiological course of the pregnancy by carrying out the necessary examinations required for this purpose,

c) to prepare women and families for childbirth, the puerperium and parenthood and to instruct and advise parents on the nutrition, care, hygiene and care of their newborn and infant,

d) recognise stressful life situations and psychosocial problems among women and their families and, if necessary, work towards the necessary support measures,

e) to provide information about the examinations required for the earliest possible detection of high-risk pregnancies or irregularities and complications during pregnancy,

f) recognise signs of irregularities requiring medical treatment during pregnancy, childbirth, the postpartum period and breastfeeding and to take the appropriate measures for medical treatment in each case,

g) to support and accompany women and families in the event of stillbirths and miscarriages as well as terminations of pregnancies after the twelfth week of gestation,

h) to care for women during labour and monitor the unborn child using appropriate clinical and technical means for monitoring purposes,

i) to attend physiological births with cephalic presentation,

j) to carry out breech births in urgent cases,

k) to transfer the woman and the newborn child to specialists for further medical treatment,

Attachment 1 to Striebich S, Bauer NH, Dietze-Schwonberg K, Grieshop M, Kluge-Bischoff A, Zyriax BC. *Academic training of midwives in Germany (part 2): Opportunities and challenges for the further development of the profession of midwifery.* GMS J Med Educ. 2024;41(3):Doc32. DOI: 10.3205/zma001687

l) to provide assistance with medical procedures while continuing the provision of midwifery care,<br>m) in an emergency and in the absence of a doctor, initiate and carry out the medically necessary interventions, in particular the manual removal of the placenta, which may be followed by a manual revision of the uterus if required, as well as<br>n) to carry out resuscitation on the woman and the newborn in an emergency,<br>o) to examine and care for the newborn and the mother after birth and throughout the postnatal period and to monitor their health condition,<br>p) to provide appropriate information and counselling on the topic of family planning,<br>q) to keep a record of the measures taken, the course of the pregnancy, the birth and the postnatal period,<br>2. carry out medically prescribed measures independently, in particular measures relating to initial care of mother and newborn child after obstetric interventions and operations, to communicate professionally with other related disciplines and work together effectively using appropriate terminology, to develop individual, multidisciplinary and interprofessional solutions, especially for abnormal pregnancy, labour and delivery, and to implement them in a team-oriented manner.

Table S2: *Competences of B.Sc. Midwives according to HebStPrV 2020 ([https://www.gesetze-im-internet.de/hebstprv/BJNR003900020.html], Appendix 1)*

I.	Independent and evidence-based support and management of physiological processes during pregnancy, birth, the postnatal period and breastfeeding. This includes the ability to recognise risks and irregularities in the woman and child and to ensure continuous midwifery care with the involvement of the necessary medical expertise.
II.	The ability to plan, organise, implement, manage and evaluate even highly complex care processes in a manner that is both efficient and effective, while also ensuring the promotion of health and the prevention of illness during pregnancy, birth, postnatally and during breastfeeding.
III.	The promotion of women's autonomy and empowerment of their right to self-determination during pregnancy, birth, the postnatal period and breastfeeding, taking into account their life situation, their biographical experiences and diversity aspects, while observing the legal obligations to act.
IV.	Communication that is both person- and situation-oriented during the support process.
V.	Shaping intra- and interprofessional action in different systemic contexts, further development of midwifery-specific care for women and their families, and participation in the development of quality and risk management concepts, guidelines and expert standards.
VI.	Reflection and justification of their own actions, taking into account the legal, economic and social framework conditions and professional ethical values and attitudes, as well as participation in professional development.
